# Exploring Convolutional Neural Network Architectures for EEG Feature Extraction

**DOI:** 10.3390/s24030877

**Published:** 2024-01-29

**Authors:** Ildar Rakhmatulin, Minh-Son Dao, Amir Nassibi, Danilo Mandic

**Affiliations:** 1 Department of Electrical and Electronic Engineering, Imperial College London, London SW7 2AZ, UK; a.nassibi15@imperial.ac.uk (A.N.);; 2 National Institute of Information and Communications Technology (NICT), Tokyo 184-0015, Japan

**Keywords:** EEG, machine learning, CNN, signal processing

## Abstract

The main purpose of this paper is to provide information on how to create a convolutional neural network (CNN) for extracting features from EEG signals. Our task was to understand the primary aspects of creating and fine-tuning CNNs for various application scenarios. We considered the characteristics of EEG signals, coupled with an exploration of various signal processing and data preparation techniques. These techniques include noise reduction, filtering, encoding, decoding, and dimension reduction, among others. In addition, we conduct an in-depth analysis of well-known CNN architectures, categorizing them into four distinct groups: standard implementation, recurrent convolutional, decoder architecture, and combined architecture. This paper further offers a comprehensive evaluation of these architectures, covering accuracy metrics, hyperparameters, and an appendix that contains a table outlining the parameters of commonly used CNN architectures for feature extraction from EEG signals.

## 1. Introduction

The beginning of investigation into the chemical processes of the brain is attributed to D. Reymond (Du Bois Reymond), who in 1849 demonstrated that the brain, like nerves and muscles, possesses electrogenic properties [1]. However, it was not until 75 years later, in 1924, that the German psychiatrist Hans Berger made the first recording of an electroencephalogram (EEG) [2]. In the years that followed, this term became widely used in medicine, creating a new scientific field known as neuroscience. In 1935–1936, Pauline and Hallowell Davis recorded the first known event-related potential (ERP) in awake individuals, and their results were published a few years later in 1939 [3]. This significantly increased the popularity of using EEG for clinical purposes. ERPs are event-related voltage changes in EEG activity that are time-bound to sensory, motor, and cognitive events. Consequently, ERP analysis is used to identify and measure specific electrical signals associated with cognitive processes, and these signals can be used to control external devices such as prostheses or computers [4,5].

**Figure 1:**
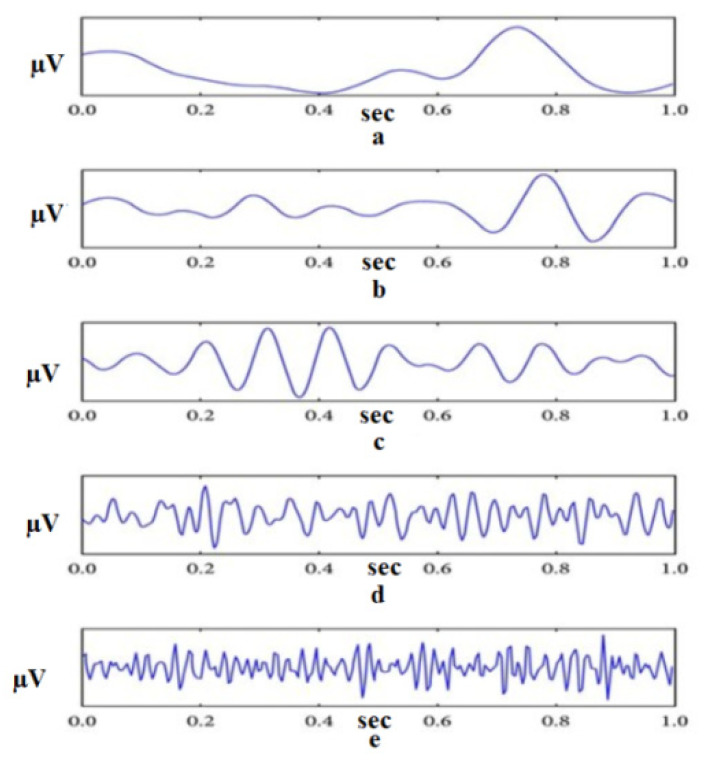
Example of EEG decomposed into its frequency bands (From **top** to **bottom**: The delta, theta, alpha, beta, and gamma frequency bands) [19].

Electroencephalogram (EEG) signals are essential for various applications and serve as a valuable tool for understanding and interacting with the human brain [6]. Notable applications include sleep monitoring, which assists in sleep pattern analysis and diagnosing sleep disorders [7]. EEG signals contribute to emotion recognition by correlating patterns of brain activity with different emotional states [8]. In motor imagery tasks, the EEG facilitates the control of external devices, influencing rehabilitation and assistive technologies [9,10,11]. Neurofeedback therapy uses EEG to regulate brain activity, offering potential benefits for conditions such as anxiety [12]. In assessing cognitive load, EEG is used to measure mental workload during tasks important to human-computer interaction and design [13]. EEG is critical for diagnosing and monitoring epilepsy, assessing traumatic brain injury, and assessing the impact on brain function [14]. These applications highlight the versatility of EEG signals across a wide range of contexts related to the human brain. In addition, the EEG is one of the key components of brain-computer interfaces (BCI), providing direct communication between the brain and external devices [15]. Different activities, such as disease diagnosis or robot control, exhibit unique characteristics that necessitate a deep understanding of the underlying physical processes. For example, the control of sleep is influenced by various factors, with the frequency component playing a crucial role [16]. During sleep, humans go through six distinct stages: wakefulness, rapid eye movement sleep, and four non-REM sleep stages, each having its own characteristic frequency [17,18], as shown in Figure 1.

EEG signals exhibit non-linear characteristics, which pose challenges in their mathematical description, as indicated by previous studies [20,21]. Furthermore, a non-linear relationship exists between the object of interest and EEG data, which standard algorithms struggle to capture. While a linear relationship does exist between electrochemical synaptic activity (expressed as current density) and EEG data in accordance with Poisson’s equation and Maxwell’s equations [22,23,24], practical applications require a focus on underlying mental or cognitive processes rather than solely the electrochemical activity of the brain. For example, emotions do not linearly correspond to brain activity. This, combined with the linear mapping of brain activity to EEG data, implies that emotions do not have a linear relationship with EEG signals.

Recent developments in CNNs have shown their prowess in handling non-linear dependencies and effectively decomposing them into their characteristic frequency components [25]. As a result, many developed architectures can operate on raw EEG data without the need for any additional processing. However, the use of CNNs poses a limitation, given their “black box” nature [26,27], whereby an output is obtained without comprehending how it occurred. Nevertheless, despite CNNs finding numerous applications across various fields, we maintain the perspective that comprehending the data processing is crucial for fully grasping the significance of their architecture [28].

### 1.1. Research Problem Statement

In existing review articles on machine learning for EEG, authors typically avoid discussing general topics like “review of EEG algorithms” due to their broad nature. Instead, they often focus on how machine learning can be applied to address specific diseases [29]. However, we believe that this approach limits the potential of machine learning and does not show how a single machine learning algorithm can be adapted to similar tasks of extracting features from EEG signals. In this manuscript, our primary goal is therefore to illustrate how CNN architectures can be adapted to suit individual needs in response to changing tasks. To achieve this goal, we aim to provide readers with a comprehensive understanding of the signal processing within a CNN and examine all aspects of the CNN tuning process as it pertains to EEG signals. We have chosen to focus on CNNs as they are one of the most promising areas in the field of artificial intelligence [30,31] and are particularly well-suited for real-time processing, which is crucial when dealing with signals that exhibit non-linear and non-stationary properties. Instead of classifying CNNs based on the tasks of their application, we have categorized them into the following groups for analysis:

- The standard implementation of CNNs
- RCNN-based architectures
- Decoder-based architectures
- Cascade Architecture.

To gain a better understanding of specific CNN architectures, it is important to have a basic understanding of signal processing. By doing so, we hope to provide readers with the necessary background knowledge to fully comprehend and appreciate the significance of various CNN architectures.

### 1.2. Machine Learning for EEG. Why CNN?

The field of machine learning is incredibly diverse, encompassing a wide range of subfields, as evident from the numerous existing classification schemes and terminologies outlined [32]. In the context of EEG, traditional machine learning algorithms, such as decision trees, support vector machines, and random forests, are commonly employed to classify EEG data based on different features, including the amplitude, frequency, and coherence [33,34]. These algorithms are widely used and have proven to be effective in various applications related to EEG analysis.

CNNs belong to the class of artificial neural networks (ANNs) and are primarily used for visual image analysis [35]. They represent a regularized version of multilayer perceptrons. Multilayer perceptrons typically refer to fully connected networks, whereby every neuron in one layer is connected to every neuron in the next layer. Researchers such as Albawi [36] and Indolia [37] have aimed to provide an understanding of CNNs and have considered general information about their structure. In a more closely related context to our topic, CNNs have been applied in medical image understanding [38] and in understanding the behavior of 3D CNNs for the diagnosis of Alzheimer’s disease based on brain imaging [39]. In this context, ANNs, including CNNs, can analyze complex non-linear relationships between EEG inputs and output classification labels [40,41]. This analysis is used to classify EEG signals based on different cognitive states, such as wakefulness, sleep stages, or various types of epileptic seizures [42,43].

CNNs can collect temporal information, automatically extract features, scale large datasets, and have the flexibility to adapt to various EEG applications. At the same time, it is often believed that for many data analysis tasks that require detection and dominance of the frequency range in EEG signals, band-pass filters may be used. However, CNNs automatically learn and extract complex features from raw input data, whereas bandpass filters can only capture simple frequency patterns.

There are numerous review articles on the topic of machine learning for EEG that provide a comprehensive assessment of various architectures. In a well-known publication, Lotte et al. [16] offered a comprehensive overview of machine learning for EEG. Later, an updated analysis was conducted after 11 years, providing insights into the field in a more modern context [44]. The updated work presented a wide range of methods for processing EEG signals but provided fewer details on feature extraction and gave insufficient attention to deep learning architectures. As a result, the article described some architectures that are not commonly used in practice, potentially causing confusion. Furthermore, significant advances have occurred in the field of neural networks over the last 5 years, which are not fully reflected in the article.

Numerous detailed reviews have explored the use of machine learning in EEG signal analysis for disease detection and other applications. For instance, Maitin et al. [45] provided an insightful review of the use of machine learning for Parkinson’s disease detection, while Rodrigues et al. [46] presented a comprehensive overview of the utilization of machine learning for the detection of alcoholism and other diseases. In addition, Rasheed et al. [47] reviewed various machine learning architectures for epilepsy detection from EEG signals, while Lucas et al. [48] explored the use of machine learning for detecting pathologies in EEG signals. In the field of emotion recognition, Bazgir et al. [49], Xiao-Wei et al. [50], and Nedelcu et al. [51] conducted thorough reviews of various techniques for removing artifacts from EEG signals using machine learning. Aggarwal et al. [52], on the other hand, focused on brain-computer interfaces and the application of machine learning in signal processing, including feature extraction and real-time processing.

There are also many smaller-scale reviews, such as “Brain-Computer Interface and Emotions” [53], along with numerous other review papers investigating the use of machine learning for disease detection via EEG signals [54,55,56,57]. Additionally, papers by Roman et al. [58] and Shedeed et al. [59] have explored the application of machine learning for signal processing in EEG analysis.

While reviews of pre-existing architectures can be helpful in determining which architecture to use for a specific case, they may not provide a complete understanding of how a convolutional neural network (CNN) is constructed or how to modify its architecture to accommodate changes in EEG measurement conditions. Although there are many review papers on the topic of feature extraction from EEG signals using machine learning, the information presented in these papers is typically not consistent in format or evaluation criteria. Therefore, in this work, we aim to provide a comprehensive guide on how to prepare, process, and build CNN architectures for EEG signals, as well as how to tune hyperparameters, estimate models, run in real-time, and address other important considerations.

In recent years, there has been a trend toward the development of new types of brain computer interfaces with the goal of making the process of measuring EEG signals more comfortable [60,61,62]. One promising approach is the use of ear-based devices for measuring EEG signals [63]. While these devices offer greater convenience, the quality of the signals they pick up is typically lower due to increased noise levels. Another alternative that has gained popularity in recent times is dry electrodes, which are also known to produce noisier signals. Consequently, the need for the development of improved machine learning algorithms for extracting useful features from EEG signals becomes even more crucial.

## 2. Signal Processing

The process of detecting EEG potentials involves measuring the potentials of electrodes placed on the surface of the head relative to the potential of an electrode on the earlobe, expressed in microvolts. The potential of the reference electrode should remain constant over time, but the scalp’s electrically active conduction system can introduce variations in potential measurements. One of the simplest solutions to address this issue is to recalculate the EEG signal drifts relative to the total averaged reference [64]. However, due to the non-stationary nature of EEG and its high susceptibility to various types of noise, especially electrical noise, the task of denouncing raw EEG data to obtain useful information poses a significant challenge [65]. In fact, the noise problem remains one of the primary obstacles to extending the application of EEG beyond the laboratory setting [66].

Pre-processing the EEG is a crucial step in preparing it for further analysis. This involves a range of techniques aimed at reducing noise and removing artifacts to ensure a clean signal is ready for subsequent steps. The initial step in this process is the removal of noise originating from external electromagnetic fields [67]. Next, motion artifacts need to be addressed, as they can have a negative impact on the EEG signal. One of the most popular tools for removing artifacts and noise from non-linear EEG signals is Principal Component Analysis (PCA) [68]. PCA reduces dimensionality and redundancy by combining original variables in a way that maximizes variance, effectively removing artifacts from the data. Independent Component Analysis (ICA) is another popular technique used for EEG signal pre-processing [69]. When dealing with multi-channel signals, ICA separates the multi-component EEG signal into its independent parts, thereby removing noise and interference caused by blinks, eye movements, heart contractions, and muscle activity. This technique has proven to be particularly effective in addressing artifacts in multi-channel EEG recordings. Canonical Correlation Analysis (CCA) is yet another technique that enhances EEG signal quality. CCA identifies linear transformations to maximize the correlations between two datasets. It has been employed to improve brain-computer interface (BCI) performance in various scenarios, such as code-modulated visual evoked potentials, steady-state visual evoked potentials, and event-related potentials like P300 and error-related potentials.

### 2.1. Signal Processing with Machine Learning

Research on EEG artifact removal methods has spanned over 55 years, yet there is still no consensus on which algorithm is optimal for a particular application [70]. Nonetheless, EEG artifact removal techniques are crucial for fully utilizing EEG data and can be implemented through both automatic and manual online and offline methods. Two of the most popular methods for EEG artifact removal are the support vector machine (SVM) [71,72] and PCA [73,74]. While these methods are commonly used, numerous other techniques are available, and several review papers aim to explore different approaches for artifact removal [75,76,77,78]. These review papers provide a valuable resource for researchers seeking to evaluate the effectiveness of different artifact removal methods. Despite the lack of consensus on the optimal algorithm, ongoing research in EEG artifact removal methods continues to enhance EEG data quality and improve analysis accuracy. The use of various techniques, including SVM and PCA, can effectively mitigate artifacts in EEG signals, enabling comprehensive utilization of EEG data in a wide range of applications.

In recent years, several researchers have explored the use of CNNs for EEG noise reduction, demonstrating promising results. In a 2020 study, Sun et al. [79] introduced CNN-1D-ResCNN, one of the first applications of CNN for EEG noise reduction. Similarly, Yang et al. [80] employed CNN and an auto-encoder, incorporating weights into an objective function to remove artifacts without compromising the EEG field signal. These studies demonstrate the potential for CNNs to effectively reduce noise in EEG signals.

Another promising approach for EEG noise reduction involves the use of recurrent neural networks (RNNs) in conjunction with CNNs. Zhang et al. [81] presented an architecture that combines CNNs and a recurrent neural network (LSTM) to eliminate dangling artifacts, naming it EEGdenoiseNet. The incorporation of RNNs in this architecture enables the network to consider temporal dependencies in the EEG signal, enhancing the effectiveness of the noise reduction process. Furthermore, Mashhadi et al. [82] successfully transformed each EEG signal into an image for input into a model designed for image segmentation tasks. This model, based on a convolutional neural network architecture known as U-NET [83], allowed for the selection of weights and filters that removed artifacts from the EEG signal, underscoring the versatility of CNNs in processing EEG data. Overall, the use of CNNs, RNNs, and other deep learning techniques shows promise in reducing noise and artifacts in EEG signals. As research progresses, these methods are likely to become increasingly effective in improving the quality of EEG data for analysis.

Despite the promising results of using machine learning techniques for EEG artifact removal, there are still some limitations prior to their widespread application. One of the primary challenges stems from the non-linear nature of artifacts, which complicates the task of isolating artifacts while preserving the valuable information within EEG signals. Another significant constraint is the substantial computing power required for machine learning algorithms, rendering them less accessible and practical for many researchers. Consequently, researchers often opt to employ machine learning to extract features from EEG signals rather than direct artifact removal. This approach allows for a hybrid model, where artifacts are eliminated in the initial layers while subsequent layers extract features. Nonetheless, the application of machine learning for EEG artifact removal remains an active area of research, with ongoing advancements in computational capabilities and machine learning algorithms expected to enhance the effectiveness and accessibility of these methods. Therefore, it is anticipated that machine learning techniques will assume an increasingly significant role in EEG artifact removal in the future.

In addition to artifacts, EEG signals can also be affected by noise stemming from external sources, such as electromagnetic interference [84], suboptimal skin-electrode contact, or low electrode quality [85]. To address these issues, researchers have developed denoising techniques aimed at eliminating unwanted noise while preserving the EEG signal’s non-linear characteristics [86].

Denoising techniques typically involve signal processing methods, including filtering, averaging, and wavelet decomposition. It is imperative to apply these methods with care to ensure that the denoising process does not distort the inherent EEG signal or remove any critical information. Therefore, researchers must diligently assess the effectiveness of various denoising techniques and select the most suitable method for their specific application. In summary, denoising constitutes a crucial step in the EEG signal processing pipeline and is indispensable for obtaining precise and dependable results in EEG experiments.

### 2.2. Frequency and Spatial Components in EEG Signals

Spectral analysis is a widely used method for extracting valuable information from EEG signals. By analyzing the power spectral density (power spectrum) of the signal, spectral analysis can provide insight into the frequency composition or the distribution of signal power over frequency. This information aids researchers in understanding the underlying neural processes responsible for generating the EEG signal and in identifying patterns that may be associated with specific cognitive states or behaviors. The power spectrum can be computed through various techniques, including Fourier transform, wavelet transform, and autoregressive modeling. These methods enable researchers to analyze the power of different frequency bands in the EEG signal, such as the alpha, beta, theta, and delta bands. By examining changes in the power spectrum over time or across experimental conditions, researchers can gain valuable insights into how brain activity is modulated by various factors [87,88,89]. Additionally, the phase synchronization method is also rooted in the frequency component. This approach measures the tendency of two time series signals to maintain a constant phase separation over a period [90,91].

The coherence method is a widely used technique for examining the relationship between EEG signals originating from various regions of the brain [92,93]. It provides valuable physiological information about functional connectivity patterns, which can enhance the performance of EEG-based biometric systems [94]. Additionally, this approach can detect alterations in the information flow between cortical areas across different frequency bands [95].

**Figure 2:**
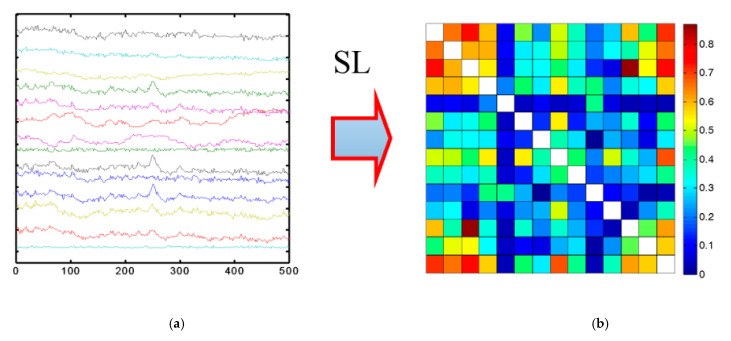
Implementation of symmetric matrix for EEG signals: (**a**) Initial data, (**b**) Symmetric matrix [97].

Moreover, the connection between these signals can also be represented using the symmetric matrix method, which illustrates the covariance of each pair of variables. The values within the covariance matrix indicate the magnitude and direction of the distribution of multivariate data within a multidimensional space. By manipulating these values, it becomes possible to extract information about how the data are distributed across any two dimensions [96]. Examples of the implementation of a symmetric matrix are illustrated in Figure 2.

The symmetric matrix can be complemented with additional methods, such as Riemannian geometry [98], to improve the performance of EEG data analysis for low-dimensional problems [99,100]. These methods enable a clearer tracing of the relationship between signals in the cerebral cortex using spatial filters. A spatial filter is an algorithm utilized for multi-channel electroencephalogram (EEG) analysis [101], often employed to extract features from EEG data based on variance. These features are then integrated into a deep neural network for classification [102].

**Figure 3:**
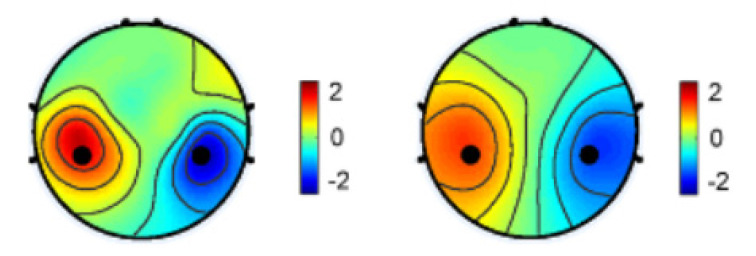
Spatial distribution of EEG power difference for the left and right hands [107].

One particularly effective spatial filter is the Common Spatial Patterns (CSPs), which has demonstrated success in extracting sensorimotor rhythms and can be employed in real-time in brain-computer interfaces. International competitions have further demonstrated the effectiveness of spatial filters in conjunction with machine learning models [103,104,105]. In addition, their application in classification problems, spatial filters can also enhance the signal-to-noise ratio in regression-based problems [106] or accentuate differences in power between various imaging conditions [107], as shown in Figure 3.

## 3. Feature Selection and Feature Extraction

EEG data are measured in microvolts, but their amplitudes can vary significantly, necessitating data scaling. The most commonly used tools for this purpose are normalization and standardization [108]. Normalization typically involves rescaling values to the range [0, 1], while standardization usually means rescaling the data to have a mean of 0 and a standard deviation of 1 (unit variance). In practice, normalization is a more frequently employed method of data preparation [109,110,111,112], though standardization is still used [113]. One notable variation on normalization is stratified normalization [114], introduced by Fdez [115], which is particularly useful for training deep neural networks to classify emotions across subjects using EEG signals. This method effectively removes between-participant variability while preserving emotional information in the data, compared to less popular methods like batch normalization [116].

Feature selection is a crucial step in enhancing the performance of machine learning algorithms by eliminating unnecessary, redundant, or noisy features from feature vectors. In EEG signal processing, statistical features such as mean, median, variance, standard deviation, and skewness are often used as the simplest features [116]. Frequency domain features (FDF) can also be computed using the discrete Fourier transform of raw EEG signals [117].

In addition to these standard methods, custom approaches have also been developed. For example, Duan et al. [29] introduced a new decision tree-based feature selection method for EEG signals, involving a feature space search and automatic selection of optimal features using a decision tree algorithm. Another approach is to utilize PCA for feature extraction from the EEG signal and subsequently employ a decision tree-based selection process to automatically select the optimal features. This method has been shown to effectively reduce the dimensionality of the EEG data while preserving important information. The EEG signal is a non-linear graph containing an extensive amount of information, with each aspect potentially representing an independent feature useful for specific functions. Therefore, feature selection is crucial in EEG signal processing to identify the most important features for a given analysis. Several survey projects have been conducted in this area [118]. It is worth noting that EEG devices can have up to 1024 electrodes, making processing all channels a computationally intensive task. To address this challenge, Alotaiby et al. [119] presented a survey of algorithms for channel selection for machine learning models. These algorithms aim to select the most informative channels while minimizing the computational burden.

Feature extraction is a critical step in EEG signal analysis, involving the conversion of raw data into numerical features that can be processed while retaining the information in the original dataset. Various popular methods are used for feature extraction in EEG signal processing, including Wavelet Transform (WT), Fast Fourier Transform (FFT), Time Frequency Distributions (TFD), Eigenvector Methods (EM), and Auto-Regressive Methods (AR), which are described in detail in, e.g., Refs. [120,121].

**Figure 4:**
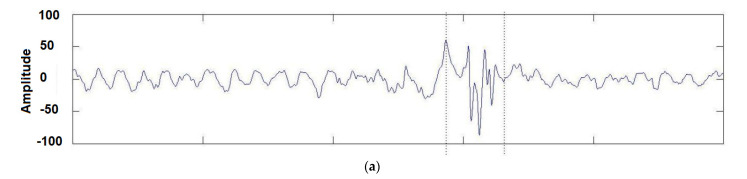

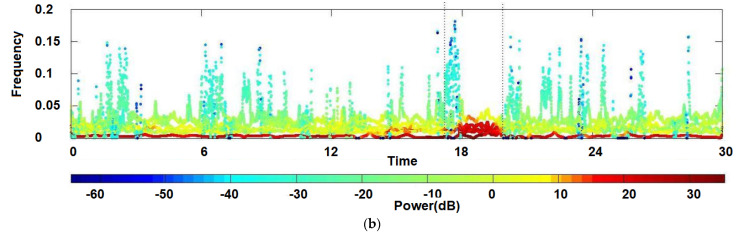
Hilbert transform of a set of real-filtered EEGs: (**a**) The analyzed time series; (**b**) The signal power plotted in the time-frequency domain [125] Once the data has been processed and transformed into numerical features, it can serve as input for machine learning models. By choosing the right feature extraction method, researchers can derive informative and meaningful features from EEG signals, thereby enhancing the accuracy and efficiency of machine learning algorithms when applied to these signals.

One well-known method for feature extraction is the Hilbert–Huang transform, a time-frequency method that decomposes EEG signals into empirical modes or intrinsic mode functions (IMF) to obtain instantaneous frequency data [122], as shown in Figure 4. Unlike the Fourier transform [123] used in harmonic analysis [124], instead of decomposing a signal into its constituent frequencies, the Hilbert–Huang transform aims to decompose data into its AM-FM intrinsic monocomponent modal functions, perceiving the locality of information. See Figure 4 for an illustration.

## 4. Datasets and Transfer Learning in EEG

Acquiring suitable datasets for training and evaluating CNN models applied to EEG data presents unique challenges compared to popular tasks like machine vision, primarily due to the inherent characteristics of biodata.

EEG recordings, in particular, are known for their inherent noise, erratic behavior, susceptibility to artifacts, and significant variability from person to person and session to session. These issues often introduce biases, confounders, and limitations that can impact the accurate estimation of the CNN architecture’s performance.

One of the primary challenges in collecting EEG datasets for CNNs is ensuring the quality and reliability of the recorded data. Various forms of noise, including environmental noise, electrode artifacts, and muscle or eye movement artifacts, can distort EEG signals and compromise the accuracy of subsequent analysis. Addressing these issues necessitates stringent electrode placement protocols, precise equipment calibration, and diligent artifact detection and removal methods. Ideally, all data should be included in datasets, but in practice, this is not always the case. Moreover, the spatial resolution of EEG signals is limited, further complicating dataset acquisition. The scalp-based nature of the EEG recording makes it challenging to precisely localize nerve sources and differentiate activities in neighboring brain regions. Proper preprocessing and data completion are crucial for CNN analysis of EEG datasets. Effective filtering techniques, artifact removal algorithms, and appropriate data segmentation methods are needed to enhance the signal-to-noise ratio and provide meaningful and robust inputs for training CNN models.

The reliability, interpretability, and performance of CNN models when working with EEG data are critical factors. This chapter will focus on available datasets and cover several important aspects, some of which are less commonly addressed.

### 4.1. Analysis of Datasets

While numerous research papers present their own datasets for evaluating machine learning models applied to EEG signals [126,127], it is worth noting that these datasets are often narrowly defined and may lack the broad recognition associated with well-established benchmark datasets, like the Microsoft-COCO datasets [128] used in machine vision tasks. Approximately a decade ago, there were popular brain-computer interface (BCI) competitions in which researchers competed to develop machine learning architectures for feature extraction from EEG signals [129,130,131]. Some parts of the datasets used in these competitions are still available. Additionally, several review papers have attempted to generalize datasets for application problems [132,133]. Additionally, datasets and architecture evaluations can be accessed through platforms such as Kaggle competitions (https://www.kaggle.com/competitions?searchQuery=EEG, accessed on 26 April 2023). However, each EEG dataset is typically unique, varying in the number of channels, electrode locations, data acquisition frequency, and other factors, making it challenging to adapt them for use with different CNN models. To address this issue, transfer learning is employed, which involves using the trained weights obtained from one dataset with another related dataset. In a review paper, Wan et al. [134] and Zhang et al. [135] conducted a search of the literature from 2010 to 2020 on the use of transfer learning in EEG decoding for brain-computer interfaces.

Another approach is self-supervised learning (SSL) [136], which can generate a dataset from the data themselves, simplifying the dataset collection process. Creating an EEG dataset using self-monitoring reduces the need for time-consuming EEG annotation [137]. In principle, SSL algorithms aim to derive everything they need from the data itself [138]. However, self-monitoring systems require a substantial amount of data, and the architecture must be efficient in terms of runtime and memory requirements. To date, SSL in EEG has been widely used in tasks such as anomaly detection in electroencephalography [139] and especially in sleep phase detection tasks [140,141,142,143]. SSL is a promising method for data label detection and has the potential to be applied to a wider range of tasks for feature extraction from EEG signals [144,145,146]. However, the application of SSL to EEG signals is not as widespread, and it is often used to identify events with prominent frequency components.

### 4.2. Overfitting in EEG Data

Overfitting in machine learning is a phenomenon in which the constructed model performs well in explaining examples from the training set but exhibits relatively poor performance when tested on new examples not included in the training set. This issue is particularly prevalent in EEG data analysis for several reasons. Firstly, the use of a large number of channels (e.g., 1024) and a high frequency (1000 Hz) results in a vast amount of data, much of which may not be relevant for practical tasks. Consequently, machine learning models can inadvertently learn patterns from noise rather than the actual events of interest within the dataset.

One of the commonly employed methods to solve the problem of overfitting is cross-validation [147,148]. Nevertheless, King et al. [149] demonstrated that cross-validation is not always a comprehensive solution for overfitting issues when dealing with EEG data, and it cannot serve as a universal method. As expected, many authors concur that one of the fundamental principles for addressing overfitting problems is to utilize a larger amount of EEG data [150,151].

To address the challenge of overfitting in CNN architectures, one approach is to reduce the number of hidden layers in the network. Zhang et al. [152] introduced a highly accurate neural network that reduces overfitting by incrementally increasing feature sizes and downsampling time series to eliminate muscle artifacts. Regularization is another commonly employed technique to prevent overfitting in EEG, as demonstrated by Zhang et al. [153] and Raduntz et al. [154]. Ying et al. [155] conducted an extensive review of contemporary methods for addressing overfitting issues in EEG.

### 4.3. Dimension Reduction of EEG Data

Reducing the sampling frequency is a common approach to mitigating overfitting, as typical sampling frequencies for reading data are around 1000 Hz [156]. However, it is important to note that upsampling can negatively impact the performance of a convolutional neural network [157], and downsampling may result in a loss of information unless it is known that the relevant data lies within a specific frequency band accessible through the Nyquist frequency. A simple algorithm for dimensionality reduction in EEG signals has been presented by Pagnotta et al. [158], but it should always be considered that lower sampling rates can lead to a reduction in model quality and connectivity estimate accuracy. Balancing the trade-off between mitigating overfitting and preserving signal quality requires careful consideration.

One of the most widely employed methods for dimensionality reduction in EEG data are PCA, which has been extensively utilized in various studies [159,160,161]. PCA reduces the dimensionality of a data set with “m” features into a subspace with fewer “n” features while retaining most of the information (or variance) from the original data set.

In addition to PCA, there are other techniques for dimensionality reduction that can be employed to prevent overestimation, including lock-boxes [162], blind signal analyses [163], pre-registrations [164], and nested cross-validation [165]. Pooling in convolutional neural networks [166,167] can also be used to reduce dimensionality. For example, Nakagome et al. [168] demonstrated that downsampling neural network-based decoders can effectively reduce dimensionality in recurrent networks. While Tang provided an overview of methods for dimensionalizing EEG signals [169], it is important to note that there are numerous techniques available, and some of them may be beyond the realm of neuroscience [170].

### 4.4. Data Representation in Different Dimensions

**Figure 5:**
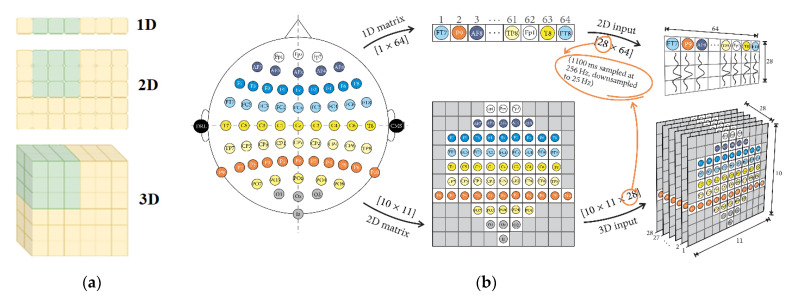
Standard implementation of data transfer for 1D, 2D and 3D formats (**a**) Chollet et al. [171] location of electrodes on the matrix according to the placement on the scalp, (**b**) Sugi et al. [172].

EEG data can be represented in various formats, depending on the algorithmic requirements and the research questions being addressed. One common representation is the 1D format, which is utilized when convolution kernels move along a single dimension, such as when analyzing EEG data over time. Another representation is the 2D format, which allows convolution kernels to move along two dimensions, facilitating the depiction of EEG data as matrices. The 3D format involves a convolution kernel convolving with the input layer, generating an output tensor. Figure 5 illustrates how EEG data can be fed into CNNs using the standard implementations for 1D, 2D, and 3D formats, with the electrode locations on the matrix corresponding to their positions on the scalp.

**Figure 6:**
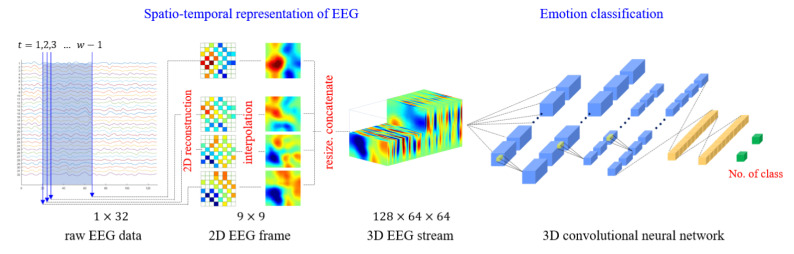
A 3D CNN for emotion recognition tasks.

Sugi et al. [172] introduced a CNN model that utilized 3D input for stimulus presentation intervals of 500, 400, and 300 ms, achieving remarkable P300 classification accuracy rates exceeding 80%. Similarly, Cho et al. [173] developed an emotion recognition method employing 3D convolutional neural networks (3D CNNs) to efficiently represent spatiotemporal features of EEG signals. Specifically, the authors reconstructed raw EEG signals as stacks of one-dimensional (1D) time series data into two-dimensional (2D) EEG frames based on their initial electrode positions. They then combined these frames with the time axis to obtain the 3D EEG stream, which they analyzed using 3D CNNs. Figure 6 illustrates these 3D reconstructions and their use in feature representation from spatiotemporal data.

Recent research has explored various formats for presenting EEG data in CNNs for classification tasks, including 1D, 2D, and 3D formats. For instance, Oralhan et al. [174] presented EEG data in all three formats for classifying visual evoked potentials in a wireless brain-computer interface system, with the 3D CNN achieving the best results at an average classification accuracy of 93.75%. While the 2D format is more commonly used in the reviewed literature, some authors have suggested that 3D formats are better suited for studying spectral and spatial information in CNNs, as well as tasks that involve time and relationships, such as word recognition tasks [175,176]. However, it is crucial to recognize that the CNN architecture remains the most significant factor in determining their performance, and there is no evident correlation between data format and classification accuracy.

## 5. CNNs for EEG

**Figure 7:**
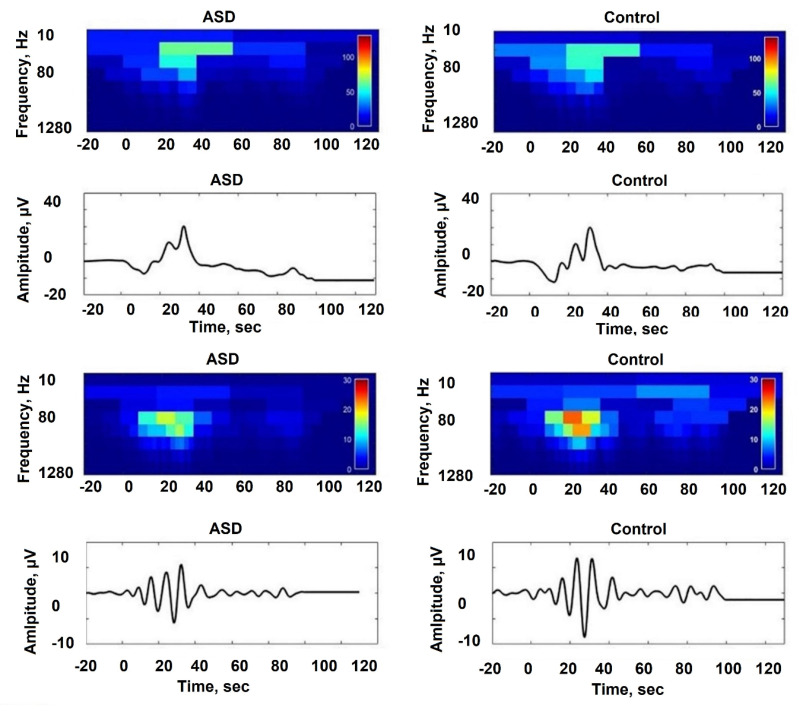
DWT waveforms for EEG data [179].

The theory of CNN has been comprehensively covered in various works, such as the book authored by Francois Chollet, the developer of the Keras framework [177]. This book explains the theory of CNNs and the mechanisms for configuring them using the Python programming language [178]. Before employing CNN algorithms in EEG signal classification, one of the primary challenges lies in the quality of the recorded signal. EEG signals encompass numerous concurrent brain activities, rendering them complex and noisy to work with. Consequently, proper pre-filtering of raw EEG signals is essential to eliminate noise and artifacts. Additionally, the selection of extracted features should be based on their correlation with the desired outcome to enhance classification performance. CNNs consist of several crucial components, including layers of convolutional filters, activations, pooling, and fully connected layers. Pooling is employed to downsample the data effectively, reducing the number of parameters and mitigating overfitting. Convolution layers extract features from the data matrix, while filters increase the depth of the feature space and help learn more levels of abstract structures. The inclusion of fully connected layers aids in learning non-linear combinations of high-level features within the output of a convolutional layer. Each EEG channel represents a non-linear plot depicting EEG data measured in microvolts, illustrating variations in magnitude over time. Frequently, the overall input signal is initially transformed into a series of 2D time-frequency images. The time series data are represented as a 1D signal on one axis, while the signal’s frequency content is represented on the other axis. The work in [179] provides a clear guide on how to convert data into heat maps, as depicted in Figure 7.

Convolutional filters are subsequently applied to each of these 2D images to extract local features, such as spikes or peaks, as shown in the heat plot in Figure 7, all of which are relevant to the task at hand. The output of the convolutional layer is then passed through one or more fully connected layers, which perform a non-linear feature transformation into the output categories.

For example, in machine vision tasks, recognizing a cat in an image involves a CNN learning to identify key visual features characteristic of cats, such as pointy ears, whiskers, and a furry body. Through a series of convolutions, activations, and merging operations, such as pooling, the CNN progressively transforms raw pixel values into a set of high-level features that capture these distinctive characteristics. After passing the image through the CNN layers, the fully connected output layer computes a set of class scores based on these features, with the highest score corresponding to the “cat” class. However, unlike searching for a cat in an image, working with EEG data are far more intricate and challenging to explain, given the data’s multidimensionality and interdependence. It is exceedingly difficult to visually identify the features requiring extraction from the graph. Consequently, the most effective approach involves the utilization of a CNN, which employs a series of trainable filters and layers. This CNN methodology enables the extraction of high-level features from the input image, facilitating the classification of these features into distinct categories among several possibilities.

### 5.1. Hyperparameters

Hyperparameters are an important component of CNN models, as they provide control over the training process and significantly impact the model’s performance and accuracy [180,181,182]. These hyperparameters encompass various factors, such as the number and size of kernels in each convolutional layer, the step size, and the size of kernels in the pooling layer [183]. While there has been extensive research on the influence of hyperparameters on CNN performance [184], the authors often provide only general guidance [185]. Determining the optimal hyperparameters for a specific model remains a challenging task, and the existing literature sometimes lacks practical insights on this matter. To address this challenge, various libraries are available for hyperparameter tuning, such as hose in Python [186], including the Scikit-learn library’s GridSearchCV and RandomizedSearchCV methods [187,188]. GridSearchCV explores all possible hyperparameter combinations to identify the best model, while RandomizedSearchCV tests random combinations of hyperparameters, making it a more efficient choice for larger datasets. Although some authors still employ RandomizedSearchCV [189], most researchers opt for GridSearchCV [190,191]. These methods prove invaluable in optimizing CNN model performance by identifying the ideal hyperparameters for a specific architecture while simultaneously reducing the risk of overfitting.

### 5.2. Kernel Size

**Figure 8:**
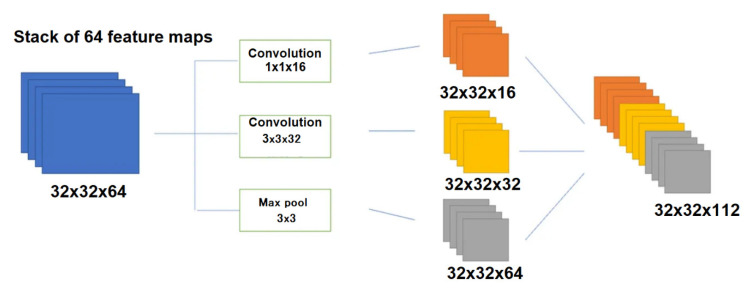
Illustration of the Inception module implemented with input data of 32 × 32 × 64 and output data of 32 × 32 × 16, 32 × 32 × 32, and 32 × 32 × 64.

Another important hyperparameter for CNN tuning is the kernel size, represented by the weight matrix used to filter the input data. Typically, small filters are employed to detect high-frequency objects, while larger kernels are utilized to identify low-frequency objects. A larger kernel size implies a less detailed examination of the data but may result in a more generalized representation of the input data. The EEG is characterized by non-linearity and non-stationarity, making it challenging to analyze comprehensively. Therefore, the selection of the kernel filter size is crucial and should be treated as if it were stationary within a specific time interval. This brings up the question of which size to choose for the kernel. Google researchers have made strides in addressing this issue by introducing a novel layer architecture called Inception [192,193]. The fundamental concept behind the Inception module is to perform multiple operations in parallel, such as combination and convolution, using filters of various sizes (3 × 3, 5 × 5, …). Figure 8 shows a convolution operation with 16 filters of sizes 1 × 1, 3 × 3. The resulting output tensor includes dimensions of 32 × 32 × 16, 32 × 32 × 32, and 32 × 32 × 64, where the last number corresponds to the number of resulting feature maps, equal to the number of filters collapsed on the image.

However, this type of model usually requires substantial computational resources, including a higher number of parameters and a longer training period [194,195].

## 6. Popular CNN Architectures for EEG

**Figure 9:**
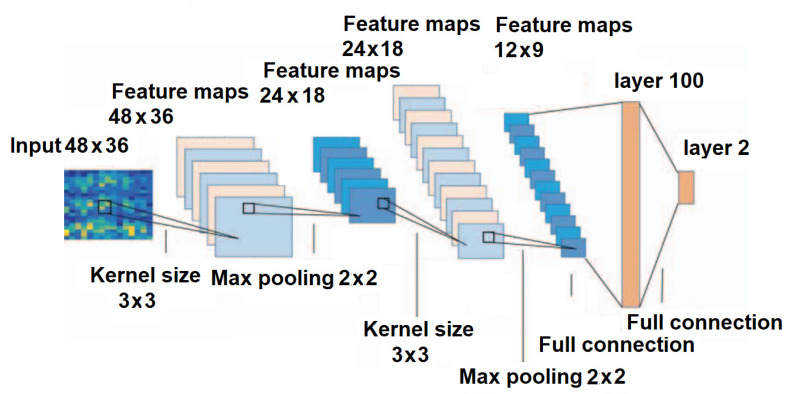
Structural diagram of the CNN architecture for motion detection [199].

The EEG signal carries valuable information at specific frequency ranges: alpha (8–13 Hz), beta (14–40 Hz), theta (4–8 Hz), delta (0.5–3 Hz), gamma (above 40 Hz), and more. Each of these frequencies has its own unique characteristics and applications [196]. Since the frequency ranges and their characteristics have been extensively studied [197,198], many researchers have decomposed EEG signals into their frequency components and then fed the data into a CNN. It is important to note that one of the advantages of a CNN is its ability to discover dependencies that may elude human observation. Therefore, it is advisable to examine the raw data and exercise caution to prevent overfitting. In light of this, Zhang et al. [199] employed the short-time Fourier transform (STFT) method for frequency decomposition of the EEG signal to detect motor activity. The resulting data were then input into a 7-layer CNN designed for classification tasks with various core layers of 3 × 3, 2 × 2, and 3 × 3. In this CNN, the last two layers were fully connected layers, comprising 100 and 2 neurons, respectively, with the SoftMax classifier, as shown in Figure 9.

**Figure 10:**
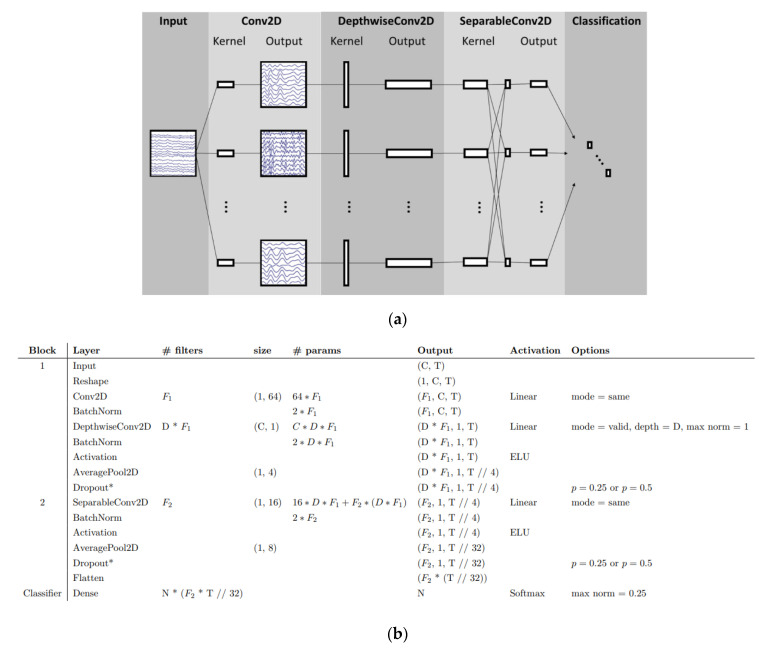
EEGNet. (**a**) Block diagram; (**b**) Model forms as proposed in [200].

The use of CNNs in analyzing EEG signals has demonstrated promising results, particularly in the detection of frequency patterns [4,33]. Some researchers continue to rely on conventional methods to decompose EEG signals before feeding the data to CNNs. While these methods may offer faster results, their accuracy needs to be thoroughly validated. Lawhern et al. [200] introduced a specialized architecture known as EEGNet, designed specifically for brain-computer interfaces. In Figure 10, it can be observed how a CNN can decompose EEG signals. The CNN starts with a time convolution to learn frequency filters, followed by a depth convolution that learns dependent frequency mass filters. The final convolution is a basic convolution that generates a temporal summary for each feature map within the sequence. This is followed by a point convolution that learns the regularity of the feature map set, with more details about the model provided. These architectures are capable of learning hierarchical features from sequential data, making them well-suited for capturing patterns in the time domain of EEG signals. The main advantage of the EEGNet is the compactness of the CNN architecture. And the model can be easily adapted for different scenarios and integrated into other architectures of ML [201].

**Figure 11:**
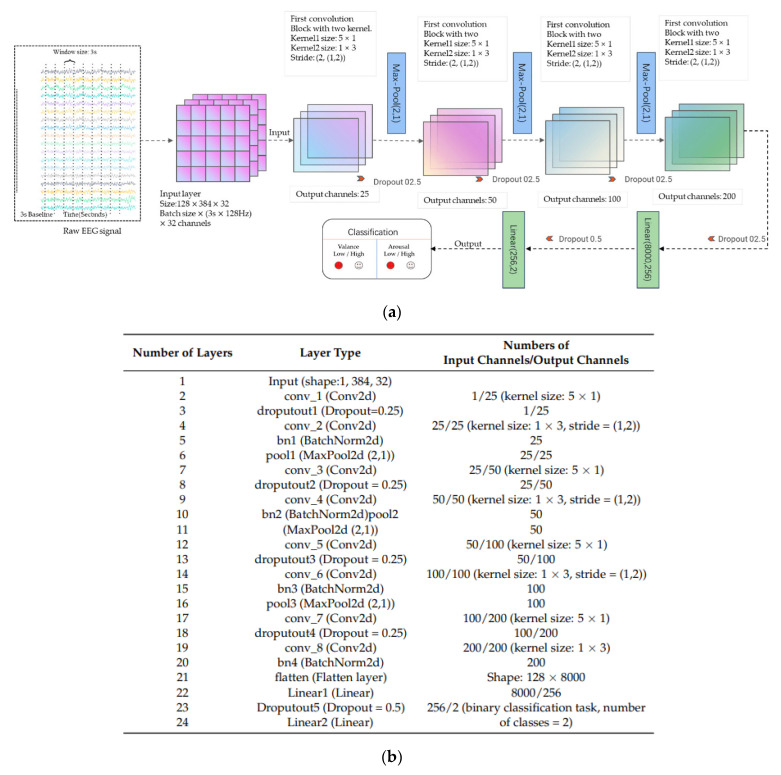
CNN architecture for emotion recognition: (**a**) Block diagram; (**b**) Model forms proposed in [202].

Wang et al. [202] introduced an 8-layer CNN designed for emotion recognition. The model’s input size was determined as width × height, where the width corresponded to 32 (representing the number of electrode channels), and the height was set at 3 × 128 = 384 (calculated as the product of the window size, 3 s, and the sampling frequency, 128 Hz). The batch size used for the model was set to 128, indicating the amount of data used in each batch. In the proposed model, Conv2 represents a multidimensional (2D) convolutional layer, Pooling2D stands for maximum 2D pooling, BatchNorm2d denotes 2D batch normalization, and Liner signifies a fully connected layer. Each convolutional layer is followed by an activation layer, Leaky ReLu. As a result, the architecture comprises eight convolutional layers, four batch normalizations, four dropout layers with a probability of 0.25, three maximum pooling layers, and two fully connected layers. The 5 × 1 convolution kernel folds the data in the temporal direction, while another 1 × 3 convolution kernel handles data folding in the spatial direction. The first three convolution blocks are connected to the maximum pooling layers at the end, and the architecture culminates in a fully connected layer utilized for emotion recognition classification. This comprehensive, lightweight model has a high degree of generalization and versatility, with an emphasis on real-time wearable applications. The visual representation of this architecture is shown in Figure 11.

**Figure 12:**
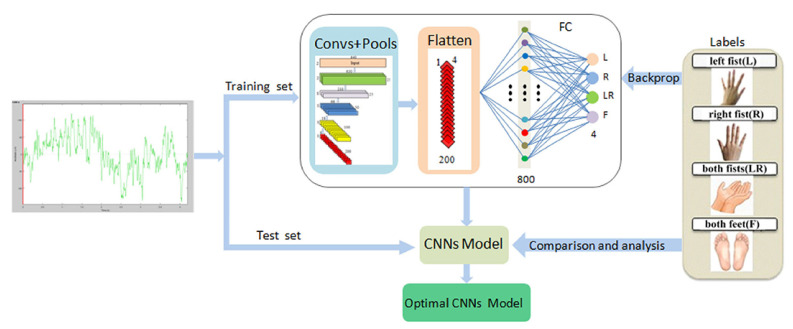
CNN structure for the task of classifying physiological activity [203].

Lun et al. [203] presented a 5-layer CNN structure designed for classifying physiological activity. The authors employed a 4-layer maximum pooling and a fully connected (FC) layer for classification. To mitigate the risk of overfitting, they incorporated dropout and batch normalization techniques. This architecture predominantly relies on 1D convolution, which is well-suited for extracting essential local features between neighboring element values of a feature vector. This model allows the decoding of raw EEG signals, providing reliability and adaptability, which simplifies the design of BCI systems for application applications. This is shown in Figure 12.

The primary challenge in EEG classification for MI tasks is its specificity. This means that each individual may exhibit unique characteristics that influence the system’s ability to correctly classify MI movement. To address this issue, multi-scale, multi-branch, or parallel architectures can be employed, enhancing the model’s generality.

The use of standard CNN architectures for EEG signal processing has demonstrated promising results in various applications, including motion image classification and seizure detection. Nevertheless, selecting optimal architecture and hyperparameters remains a task that is inherently tailored to the specific EEG signal and classification objective.

### 6.1. Architectures with Encoders and Decoders

In the context of EEG signal classification, it is common to utilize encoders and decoders to transform data into scales more suitable for CNNs [204]. The primary objective of these encoders is to reduce the dimensionality of the original feature space. Unlike PCA techniques, decoders are typically integrated into the CNN architecture and not used as a separate component of the data preparation process. Several studies have demonstrated that autoencoders outperform PCA in preparing data for CNNs [205,206]. By leveraging the CNN training procedure, autoencoders can effectively capture the salient features of objects, facilitating the recovery of the original sample objects.

Decoders are frequently employed in Generative Adversarial Networks (GANs) for various applications, including image generation [207,208]. More recently, GANs have been extended to time-series data [209], yielding promising results. Successful applications of the GAN architecture include generating synthetic data for use in LSTM networks [210], removing noise from data [211], and detecting sleep stages.

Supervised and unsupervised CNNs serve different purposes in the analysis of EEG (electroencephalography) data. Supervised CNNs are employed in EEG classification tasks where output labels are known. The networks require labeled training data to learn the relationship between EEG inputs and corresponding output labels. Supervised CNNs find applications in various domains, including emotion recognition, seizure detection, and sleep staging within EEG analysis. In contrast, unsupervised CNNs are utilized to explore EEG data, extract patterns, and identify underlying structures for research purposes. These networks are designed to learn the internal structure of EEG data without the need for labeled data. Unsupervised CNNs can be suitable for tasks such as clustering, dimensionality reduction, and anomaly detection. Autoencoders represent a specific type of unsupervised CNN used for studying EEG characteristics. The network is trained to recover input data at the output layer, with a bottleneck in the middle that learns a compressed representation of the input data. The learned features can subsequently be used for classification tasks. Therefore, supervised CNNs are employed for EEG classification tasks, while unsupervised CNNs are utilized for data exploration and feature learning.

### 6.2. Recurrent Neural Networks

**Figure 13:**
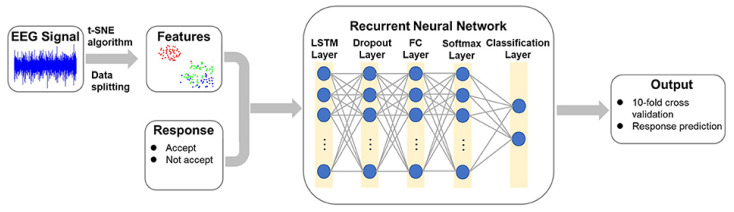
RNN based algorithm for predicting decision behavior [213].

Recurrent neural networks (RNNs) are well-suited for analyzing EEG data due to their ability to capture temporal dependencies, which are crucial for understanding brain dynamics [212]. The cyclic connections in RNNs allow the network to maintain an internal state or memory that can be updated with new data. This memory enables the network to process sequential data, such as EEG signals, while preserving temporal relationships between the data points. However, RNNs face challenges with vanishing gradients when dealing with long-length data. This issue has been addressed with the Long Short-Term Memory (LSTM) architecture [212]. Ma et al. [213] developed an architecture for predicting decision-making behavior from EEG signals using the t-SNE method. This method employs a stepwise iterative approach to represent the original data in a low-dimensional manner while preserving information about its local neighborhood. The architecture includes the t-SNE algorithm for feature extraction from EEG signals in the first stage and a recurrent neural network with a LSTM layer for predicting decision behavior in the second stage, as shown in Figure 13.

**Figure 14:**
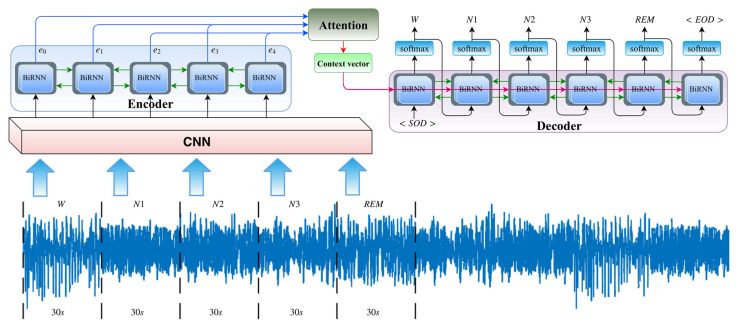
CNN architecture for automatic estimation of sleep stages [214].

LSTMs, as well as CNNs, are applicable for sleep stage detection. Mousavi et al. [214] implemented a CNN with two sections for extracting temporal and frequency information. The input signals consist of a sequence of 30 s EEG epochs, and the output data represents the corresponding stages or classes. The encoder processes the input sequence, while the decoder computes the category of each individual channel of the 30 s EEG input sequence. The encoder consists of long short-term memory (LSTM) blocks that capture complex and long-term short-term contextual dependencies between inputs and targets, as shown in Figure 14. This algorithm proposed new approaches to calculating losses, which helped reduce the impact of the class imbalance problem and improve sleep stage detection performance. These LSTM blocks address non-linear dependencies across the entire time series when predicting the target.

**Figure 15:**
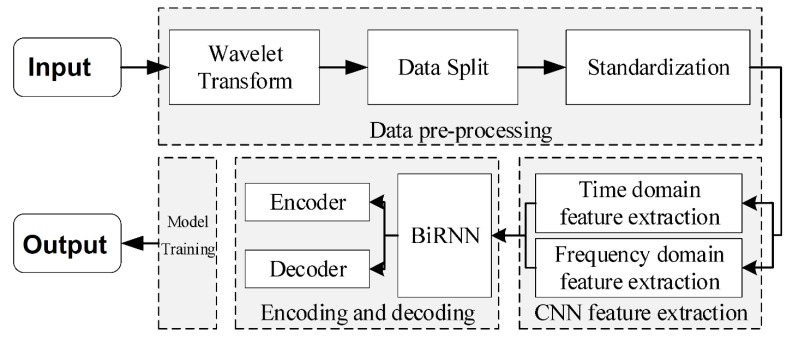
Block diagram based on BiRNN for sleep stage detection [215].

Fu et al. [215] introduced an architecture that employs a bidirectional recurrent neural network (BiRNN), consisting of an encoding and decoding module, for sleep phase detection. This approach combines time- and frequency-domain feature extraction using a CNN to expand the feature extraction domain while preserving the original EEG feature information. Time series information is extracted using the BiRNN encoding-decoding module, and automatic sleep stage discrimination of the EEG signal is performed using the SoftMax function. The block diagram of the network is shown in Figure 15.

**Figure 16:**
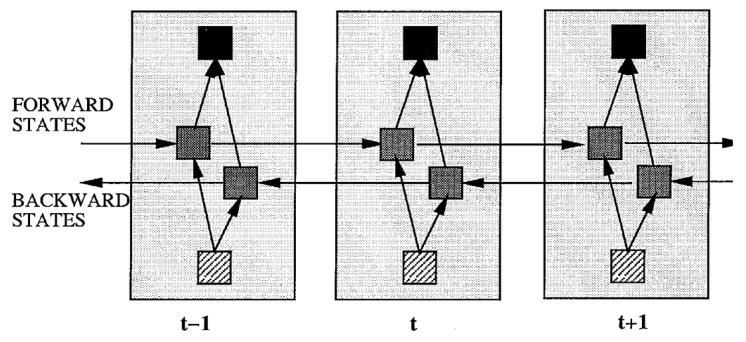
Structuring the BIRNN schema shown unfolded in time for three time steps [216].

In recent years, BiRNN has gained popularity for predicting speech from EEG signals. Schuster et al. [216] introduced the BiRNN architecture in 1997 as an extension of a RNN to a bidirectional recurrent neural network (BRNN), as shown in Figure 16. The authors showed that in the task of extracting a feature from EEG signals, the BRNN structure leads to better results than other ANN structures, while the training time for BRNN is approximately the same as for other RNNs.

Unlike the standard RNN, the BRNN divides neurons into two directions: one for the positive time direction (forward state) and the other for the negative time direction (backward state).

### 6.3. Cascaded Architecture

Many papers exist in which the authors have presented architectures that combine multiple architectures to solve a task, often referred to as a “cascaded architecture”. The advantage of this approach is that it allows for the use of, for example, parts of an architecture designed to extract frequency patterns with another part of the CNN designed for extracting spatial filters. For instance, Altuwaijri et al. [217] employed the EEGNet architecture in the first stage to work on the frequency components of the signal, as described above for image signal classification. They included a block for altering the data dimension, referred to as Squeeze–Excitation, and only in the final part of the classification task did they use a few layers of custom CNN architecture.

**Figure 17:**
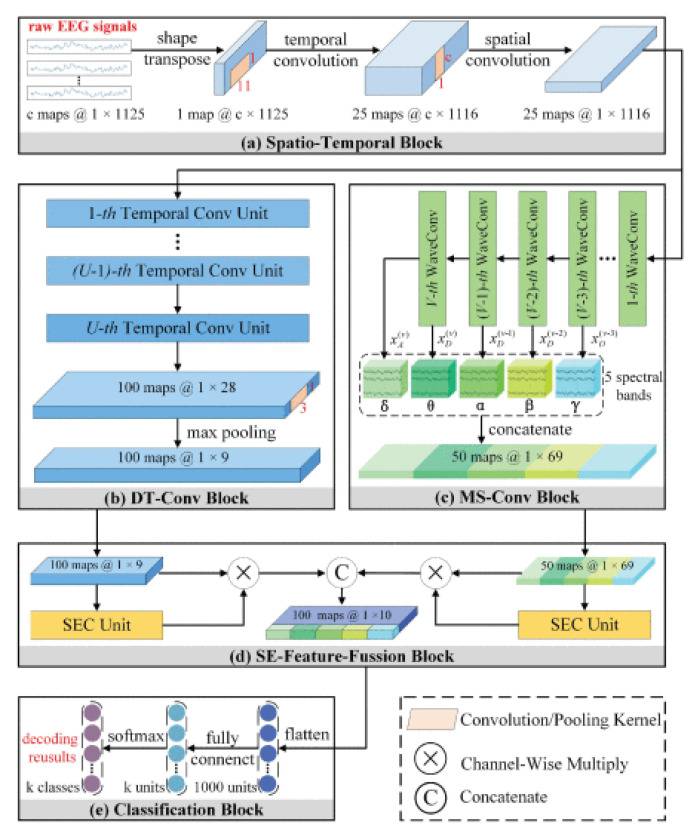
Structural diagram of a combined architecture for motor imaging classification problems [218].

Li et al. [218] proposed a solution to the motion classification problem by introducing the Temporal-Spectral Fusion of Squeeze and Excitation Functions (TS-SEFFNet). In this combined architecture, a deep temporal convolution block (DT-Conv block) was used to extract multivariate temporal representations from raw EEG data alongside a parallel multispectral convolution block (MS-Conv block). The use of multilayer wavelet convolutions enabled the extraction of information regarding the spectral component of the signal. A feature fusion block (SE-Feature-Fusion block) was employed to merge deep temporal and multispectral data into complex merged feature maps. Experimental results confirm that this architecture can effectively decode EEG, which can be considered a powerful tool for MI-EEG-based BCI. Finally, for motion classification, a classification block was utilized, as illustrated in Figure 17.

Several papers exist in which authors have presented complex combined deep architectures for the purpose of extracting features from EEG signals. These models are particularly intriguing because they are composed of distinct blocks, each of which can be adapted for use in a customized architecture. For example, Kostas et al. [219] employed a self-directed learning model for speech recognition from EEG signals. The model utilizes a multilevel convolutional feature encoder consisting of multiple blocks. Each block includes time convolution, followed by level normalization and a GELU activation function. The raw signal entering the encoder is normalized to have a mean of zero and a unit variance. The total encoder step determines the number of time steps provided to the converter. The output of the feature encoder is then directed into the context network, which follows the transformer architecture. This context network incorporates a convolutional layer for encoding absolute position information. Finally, the authors append the convolution output, followed by GELU, to the input and then apply layer normalization.

## 7. Details of CNNs in the Context of EEG Signals

The primary building blocks of CNNs include convolutional layers, subsampling (pooling) layers, activation layers, and fully connected layers. As part of EEG signal processing, we will consider hyperparameters, activation functions, and loss functions.

The loss function, in the theory of statistical decision-making, characterizes the losses incurred due to incorrect decisions based on observed data. Machine learning inherently revolves around optimization, and as in any optimization problem, we need to determine how far our predictions deviate to make the necessary adjustments. Loss functions take predictions and compare them to actual values or data labels, providing an error metric. Loss functions are fundamental components of any architecture and have been well-studied [220]. Thiyagarajan et al. [221] utilized a triplet-based loss function for clustering EEG data in their CNN, while Zhang et al. [222] applied the central loss function to improve the deep learning performance for EEG signal classification. Zhao et al. implemented focal loss for EEG-based seizure detection using a linear graph convolution network with focal loss [223]. Luo et al. [224] for EEG signal reconstruction using GAN with Wasserstein distance used temporal-spatial-frequency loss. This TSF-MSE-based loss function reconstructs signals by calculating MSE based on time series characteristics, general spatial structure characteristics, and power spectral density characteristics. Several researchers have introduced their custom loss functions, which, however, tend to be specialized and challenging to adapt to other architectures [225,226,227]. Brophy et al. [228] used a custom loss function to improve the denoising of electrode motion artifacts in ECG using convolutional neural networks. The choice of the optimal loss function for an architecture designed to work with EEG signals remains an ongoing challenge. Commonly used approaches include Mean Normalized Error (MNE) for extracting frequency patterns and Softmax loss for extracting spatial patterns.

Optimisers play a crucial role in CNN training, aiding in achieving increasingly accurate predictions. Optimizers determine the optimal set of model parameters, such as weights and biases, so that the model performs best for a given problem. The gradient descent algorithm [229,230] is a widely used optimization technique. A review of the papers showed that Adam is a commonly employed optimizer for both classification and prediction in the EEG field.

One of the stages in the development of a CNN is the choice of the activation function of the neurons. The type of activation function largely determines the functionality of the architecture and the method of training the model. The classic backpropagation algorithm [231] works well for CNNs with a few layers but encounters challenges as network depth increases, notably due to the problem of gradient attenuation [232]. The attenuation of gradients refers to the diminishing or vanishing of gradient values as they are propagated backward through the layers during the training process. In the context of the classic backpropagation algorithm, the attenuation of gradients can impede the effective updating of the network’s weights, especially in deep architectures. As the error propagates from the output layer to the input layer, the current result is multiplied by the derivative of the activation function at each layer. Different activation functions are employed in fully connected layers (FC), with the Rectified Linear Unit (ReLU) activation function being a common choice for neural network layers, especially for tasks in the frequency domain and spatial problem-solving, such as those addressed by Softmax. Dubey et al. [233] wrote about activation functions in the deep learning field; Mehta et al. [234] considered activation functions in the context of CNN; and Hao et al. [235] considered function activations more locally for EEG signal classification. In machine learning problems, metrics are used to evaluate the model quality and compare different algorithms. Understanding these metrics is crucial, as their values are used to evaluate the developed architecture. Choosing the right metrics is essential to avoid misinterpretation of the work of the CNN architecture. Carvalho et al. [236], R. Padilla et al. [237], and Saeedeh Ziyabari et al. [238] considered metrics in the context of machine learning, CNNs, and EEG signal processing.

## 8. Progress in Hardware

Laboratory equipment for non-real-time EEG data analysis faces no issues with computing power [239]. However, in recent years, there has been a growing interest in brain-computer interface devices that operate in real-time and are not highly powerful. Simultaneously, affordable consumer-grade EEG devices based on microcontrollers can measure EEG signals with the same quality as laboratory equipment [240,241]. This necessitated the implementation of machine learning algorithms directly on the microcontroller. The concept of Edge AI enables the utilization of machine learning algorithms directly on chips like Kneron, Kendryte, K210, and RISC-V [242,243]. For example, Fang et al. [244] employed Edge AI in a system-on-chip design for an EEG-based real-time emotion recognition task. TensorFlow introduced a platform for machine learning on embedded devices, known as TinyML [245], which gained popularity for microcontrollers [246,247,248]. STMicroelectronics has introduced a framework, X-CUBE-AI, for implementing machine learning algorithms on STM32 series microcontrollers [249,250,251].

Wang et al. [252] presented an EEGNet-based motor visualization brain-computer interface for low-power edge computing. To implement the EEGNet model on the ARM Cortex-M family of microcontrollers, Wang et al. [252] downsized the input feature map by reducing temporal and spatial dimensions and narrowing the time window, which relaxed the memory requirements.

Mezzina et al. [253] developed an Embedded convolutional NN (E-CNN) using two 1D convolutional layers, an intermediate batch normalization step to counter data covariate shift, and two dropout sections to mitigate overfitting phenomena. The batch size for the stochastic gradient descent was set to 128, and the optimal number of epochs to prevent overfitting was set to 50. The model was tested on STM32 microcontrollers with quantization crosses.

As the market for embedded devices continues to grow, along with the growing scope of EEG data, the significance of machine learning architectures for real-time EEG signal processing and feature extraction in embedded systems is becoming increasingly relevant.

## 9. Conclusions

In this paper, we have addressed the design of CNNs for custom tasks in the field of feature extraction from EEG signals. Our analysis encompasses several popular algorithms and explores the data preparation and hyperparameter tuning processes. One limitation in evaluating the models we have reviewed is that different datasets were used by the authors for their assessments. In neuroscience, comparing the effectiveness of models involved in different tasks is not as straightforward as in domains like computer vision, which employ widely recognized datasets such as COCO [254]. For this reason, we have provided a table in Appendix A detailing the parameters for different architectures. This table will help those who are starting to develop a custom architecture and will allow, at the initial stage, the selection of hyperparameters and the determination of the structure of the created architecture.

Recurrent neural networks, particularly LTSM, have found extensive application in event prediction from EEG signals. Encoders and decoders, serving as alternatives to PCA methods, have become effective in reducing dimensionality. These blocks are already part of the CNN architecture, and their placement within the network hierarchy depends on the architecture type.

Popular architectures can be categorized into two application areas: (a) identification of frequency patterns (e.g., sleep, emotions) and (b) spatial analysis, usually used for prediction tasks (e.g., motor imagery, speech). Standard implementations of CNNs are well suited for extracting frequency components, as evidenced by numerous papers with minor architectural and hyperparameter variations. Models based on recurrent neural networks, in particular LTSM, have been widely applied to the task of event prediction from EEG signals. Encoders and decoders, serving as alternatives to PCA methods, have become effective in reducing dimensionality. These blocks are already part of the CNN architecture, and their placement within the network hierarchy depends on the architecture type. The block architecture has shown that different model blocks can be easily implemented into new models, and the EEGNet model has become particularly popular in this direction, often being used as the first block for the task of decomposing the EEG signal into frequency components. While some papers use data preparation methods like PCA and ICA to enhance classification accuracy, many others work with raw data. CNNs exhibit an advantage in classification and prediction tasks, but one drawback when working with EEG signals is their limited generalizability, unlike the machine vision domain. In the future, we expect to see more approaches employing LSTM and more complex cascade models, with a new framework developed that will allow resource-intensive CNNs to run on hardware without large computing power.

In upcoming research, we plan to extend this article by implementing different CNN architectures (e.g., Similarity Learning Network, Multi-task learning) on the same dataset to facilitate a more direct comparison of their effectiveness. We will also consider well-established CNN architectures implemented outside the field of neuroscience. CNNs are more advanced in machine vision tasks and tuned for object detection tasks, yet the popular Yolov model [255], implemented for machine vision tasks, has also been used to extract features from EEG [256]. Therefore, it is logical for neuroscientists to look for new architects beyond the EEG domain. The accuracy of feature extraction from EEG data depends not only on the CNN architecture but also on various external factors, such as the number of EEG channels. More electrodes can provide enhanced spatial resolution, aiding in localizing neural activity and producing ERP. High-quality electrodes are effective in reducing electrical noise, and optimizing impedance matching between the electrodes and the scalp further improves the signal-to-noise ratio. Additionally, advanced data analysis techniques like machine learning and multivariate analysis can reveal subtle patterns in EEG data that are difficult to detect using traditional analysis methods. Combining EEG with other neuroimaging techniques, such as fMRI or MEG, can provide additional information about the neural processes underlying ERP.

## Appendix A

**Table A1:** Applying CNN to Feature Extraction from EEG Signals.

№	Tasks		Dataset	CNN	Learning Type	Steps	Structure	Optimization	Activation Function	Function Loss	Evaluation Metrics	Framework	Ref.
**1**	Classification task	Sleep stage annotations	Physionet Sleep-EDF dataset	SleepEEGNet	Supervised	Decomposition of data into frequency components and subsequent classification	2DCNN and BiRNN	RMSProp optimizer	ReLU	lMFE	k-fold cross validation. overall accuracy, precision, recall (sensitivity), specificity, and F1-score.	Python 3.7–3.10, TensorFlow 2.8	[256]
**2**		Emotion recognition	DEAP dataset 39	EEG-Based Emotion Recognition Using a 2D CNN	Supervised	Decomposition of data into frequency components and subsequent classification	2D CNN	Particle Swarm Optimization	LeakyReLU Outpit—Softmax	Cross Entropy	85%	Python 3.7–3.10	[214]
**3**		Motor Imagery Signals Classification	BCI Competition IV 2a (BCI-IV2a), High Gamma (HGD)	MBEEGSE	Supervised	MBEEGSE architecture. Divided into three branches, each with EEGNet and SE block	EEGNet and Squeeze-and-Excitation (SE) Block	Adam optimizer	Softmax	Cross Entropy	70%	Keras 3.0.4, Python 3.6, 3.7, 3.8, 3.9	[202]
**4**		Motor Imagery EEG Decoding	BCI Competition 2008 IV 2a Dataset High Gamma Dataset: (HGD)	TS-SEFFNet	Supervised	First, the deep temporal convolution block (DT-Conv block). Second, multispectral convolution block (MS-Conv block) is then run in parallel using multilevel wavelet convolutions. Finally, block (SE-Feature-Fusion block) displays the depth-time and multispectral features into complex pooled feature maps that extract the feature responses across channels.	DT-Conv block, MS-Conv block, SE-Feature-Fusion block	The Optimization Steps of the Proposed TS- SEFFNet Method	Softmax	Custom loss function	93.25%	Torch 1.4, Python 3.8	[217]
**5**		Sleep stage annotations	Physionet Challenge dataset	Self-supervised learning (SSL)	Unsupervised	The first step is a sampling process by which examples are extracted from the time series S (EEG recording). The following describes a learning process where sample examples are used to train the feature extractor end-to-end.	Relative positioning (RP). Temporal shuffing (TS), Contrastive predective coding (CPC)	Adam optimizer	Rectified Linear Unit (ReLU)	Cross-entropy loss function	72.3%	Torch 1.4, Python 3.9	[218]
**6**	Prediction task	EEG Imaginary Speech Recognition	Kara One database	-	Supervised	a CNN containing two convolutional layers with 64 and 128 filters connected to a dense layer with 64 neurons for input signal spectrum of a 0.25 s window	2D CNN	Adam optimizer	Linear	Categorical cross-entropy	37%	-	[257]
**7**		EEG-Speech Recognition	Custom dataset (not available)	-	Supervised	ResNet18/50/101 with 2 layers of managed recurrent units—Gated Recurrent Unit (GRU). And after that ResNet18 operation are fed to the input of a recurrent neural network containing 1024 hidden GRUs.	CNN and RNN	Adam optimizer	Softmax	-	85%	-	[258]
**8**		EEG speech recognition	Custom dataset (not available)	-	Supervised	The architecture used includes the already trained VGG Net CNN design and the target CNN design, while the already trained VGG Net CNN design extracts global features for general image classification work, and the target CNN design aims at efficient and accurate categorization of EEG signals using already trained Model VGG Net CNN.	Deep Residual–encoder–based VGG net CNN	-	Softmax	Softmax cross-entropy	95%	-	[259]
**9**		Seizure prediction	CHB-MIT and Kaggle	-	Supervised	a hybrid network that can combine the additional benefits of CNN and Transformer. The CNN is used to extract local information that contains two 3 × 3 convolutions with stride 1 and another 3 × 3 convolution with stride 2 to reduce the size of the input features. Each convolutional layer is followed by a GELU activation and a batch normalization (BN) layer. The model has two stages for extracting multiscale features from the EEG spectrum. Each stage consists of a set of Transformer blocks applied to extract long-term dependencies.	CNN and transformer	Adam	Softmax	Cross-entropy	95%	Torch 1.4, Python 3.8	[260]
**10**		Predicting Human Intention-Behavior	BCI competition IV Dataset 2b	-	Supervised	The multi-scale CNN model has seven layers, which are one input layer, two convolutional layers, one max-pooling layer, one multi-scale layer, one full connection layer and one softmax output layer. The input layer in the multi-scale CNN model is fed with a time-frequency image with the size of 40 × 32 × 3 after EEG signals are preprocessed by STFT	Multi-Scale CNN Model	-	Linear	Cross-entropy	73.9%	Python 3.8, Keras 3	[261]
**11**	Artifact Removal	EEG Artifact Detection and Correction	Costum dataset, not available	-	Unsupervised	modification of a feed-forward neural network that uses weight sharing and exhibits translation invariance. Learning in the CNNs operates on the same principle as a traditional feed-forward neural network where an error from output layer is back-propagated through the network and weights of the network are proportionally updated to the gradient of error.	CNN	Adam	-	Cross-entropy	-	Python 3.9, Keras 3	[262]
**12**		Remove Muscle Artifacts from EEG	EEGdenoiseNet	-	Supervised	CNN for myogenic artifact reduction contains seven similar blocks. In each of the first six blocks, two 1D-convolution layers with small 1*3 kernels, 1 stride, and a ReLU activation function are followed by a 1D-Average pooling layer with pool size equal to two. In the seventh block, two 1D-convolution layers are followed by a flatten layer.The network gradually reduce the EEG signal sampling rate by the 1D-Average pooling layer.	CNN	RMSprop	ReLU	mean squared error (MSE)	-	Python 3.10, Tensorflow 2.8	[263]
**13**		Denoise EEG signal from artifacts	EEGdenoiseNet	MultiResUNet3+	Supervised	Net3+ consists of full-blown pass-through connections that aggregate connections between encoders and decoders and internal connections between decoder subnets. Instead of directly combining the encoder and decoder functions, the encoder functions go through several convolutional levels with residual connections	CNN, encoders	Adam	Rely	mean squared error (MSE)	-	-	[152]
